# Molecular epidemiology and viral load of HCV in different regions of Punjab, Pakistan

**DOI:** 10.1186/1743-422X-11-24

**Published:** 2014-02-10

**Authors:** Suliman Qadir Afridi, Muhammad Muddassir Ali, Furqan Awan, Muhammad Nauman Zahid, Irfan Qadir Afridi, Sara Qadir Afridi, Tahir Yaqub

**Affiliations:** 1 Quality Operations Laboratory, UVAS, Lahore 54000, Pakistan; 2 Institute of biochemistry and Biotechnology, UVAS, Lahore 54000, Pakistan; 3 Department of Epidemiology and Public Health, UVAS, Lahore 54000, Pakistan; 4 Ideal Laboratories, Lahore 54000, Pakistan

**Keywords:** Hepatitis C virus, Genotype 3a, Pakistan, Viral load

## Abstract

**Background:**

Hepatitis C virus (HCV) is highly infectious pathogen which is responsible for causing Hepatitis around 200 million individuals worldwide. In Pakistan, 4.7% of HCV prevalence has been reported and HCV genotype 3a has been found to be the major source of infection in Pakistan but still there is lack of information on distribution of HCV genotypes and viral load in various geographical regions of Pakistan. Therefore, current study was designed to determine distribution of HCV genotypes as well viral load in different areas of Punjab province of Pakistan.

**Findings:**

A total of 995 serum samples were taken from those individuals in which antibodies against HCV were detected through ELISA, from different regions of Punjab i.e. Lahore 317(31.85%), Faisalabad 70(7.03%), Gujranwala 129(12.96%), Gujrat 106(10.65%), Sialkot 94(9.44%), Sargodha 60(6.03%), Mandibaha-ud-din 135(13.56%), Jhang 86(8.64%). Qualitative PCR was performed to determine viral load and genotyping was performed using Nested PCR. Chi-square test was used to determine the age and sex-wise prevalence of HCV. Out of 995 samples, 888 samples were found positive for HCV RNA. In all regions, genotype 3a showed highest prevalence (82.81%) followed by genotype 1 (3.41%), mixed genotypes (2.41%), genotype 2 (0.50%), genotype 5 (0.1%) and unclassified genotypes (10.75%). Viral load in 29.5% patients infected with genotype 3a was less than 600,000 IU/mL, while it was between 600,000-800,000 IU/mL in 27.9% patients and 25.22% patients had more than 800,000 IU/mL viral load.

**Conclusion:**

HCV genotype 3a is the most prevalent genotype in various regions of Punjab. Viral load of HCV patients in these different regions of Punjab are reported for the first time. Moreover, based upon these results the Patients having viral load below 800,000 IU/mL would be expected to show better response of anti-HCV therapy.

## Findings

### Background

Hepatitis C virus (HCV) is a globally distributed human pathogen that has affected around 200 million individuals. It is a principle cause of chronic liver disorders including liver cirrhosis, liver fibrosis and hepatocellular carcinoma. HCV is a positive-sense single-stranded RNA virus belonging to genus *Hepacivirus,* the member of *Flaviviridae* family, with characteristic of genetic heterogeneity [1]. HCV is an enveloped virus that contains small genome of about 9,600 nucleotides [2]. Due to genetic variability, there are 6 extensively identified genotypes and each genotype has difference of 30%-35% in its nucleotide site sequence from others [1,3]. Each genotype contains several subtypes having more than 75% nucleotide sequence similarity among them [4]. In comparison, more than 95% of the virions infecting one patient are similar [5]. HCV genotype/subtype identification is crucial for clinicians when it comes to choosing a therapy because these genotypes have been reported to exhibit different responses to prescribed anti-viral therapies and require varying duration and doses of therapy [6]. The transmission of HCV is on average six times more likely than HIV after a percutaneous exposure [7]. The distribution of virus is worldwide but some strains are particularly detected in specific geographical regions. HCV genotypes 1a, 1b, and 3a are prevalent “epidemic” strains all over the world [7]. During 20^th^ century above mentioned strains spread quickly, probably through infected blood-products and use of injectable drugs, and possess comparatively less genetic variations. On the other hand, other HCV strains have high levels of genetic variation but are confined to specific geographical areas [8]. Genotypes 1, 2 and 4 are confined to certain regions of Africa and the Middle East, whereas genotypes 3 and 6, divergent endemic strains, are detected in numerous localities of Southeast Asia [9]. The present study was designed to find different genotypes and viral load of HCV in different districts of Punjab province of Pakistan.

## Methods

A total of 995 blood samples were collected from those individuals in which antibodies against HCV were detected through ELISA, to find out their viral load and genotyping situated in different districts/ towns of Punjab, Pakistan. In order to fulfill the legal and ethical requirements, this study got approval by Ethical Committee Bacteriologist to Government of Punjab health department (No. 1029/Bact.). Written consent was taken to record the age, district, and phone and complete address of the patients. Each blood sample, subjected to get serum, was centrifuged at 3000 RPM for 5 min. Serum obtained were labeled and stored at − 20°C till extractions begin.

Extraction of RNA was done using Qiagen kit (Invitrogen, Corp., California; USA). The cDNA of 5’NCR was synthesized using 100 units of Moloney Murine Leukemia Virus (MMLV) reverse transcriptase enzyme (RTEs) (Invitrogen, Corp., California USA) with 5 pM of outer antisense primer. HCV RNA was quantified as the protocol described by Ali et al., 2011 [10]. Two rounds of PCR amplifications were done (first round PCR and Nested PCR) with two units of Taq DNA polymerase enzyme (Invitrogen, Corp., California USA) in a volume of 20 μl reaction mix. Different allele-specific primers were used for the detection of HCV genotypes [10]. Chi-square statistics using SPSS version 16.0 (IBM Corporation 2008) was used to analyse the genotype prevalence and distribution among genders and all age groups. P value < 0.05 was considered significant.

## Results

**Figure 1:**
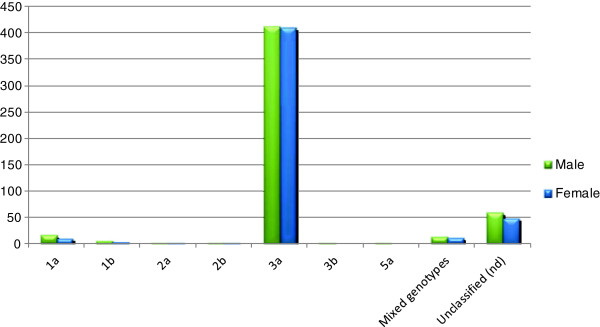
Prevalence of HCV genotypes in different genders.

Different HCV prevalent genotypes and viral load between both genders in different geographical regions of Punjab province is shown in Table 1 and Table 2. Of 995 samples, 511 (51%) samples from males and 484(49%) from females were declared as HCV positive that showed nearly equal representation between both genders. Among these 995 samples, 888 (89.25%) samples were detected on the basis of specific PCR fragment while 107 (10.75%) samples remained unable to demonstrate a specific genotypic band. Genotype 3(82.81%) was highest in both genders followed by genotype 1 (3.41%), mixed genotypes (2.41%), genotype 2 (0.50%), genotype 5 (0.1%) and unclassified genotypes (10.75%), (Figure 1). Among the sub-genotypes, the most detected sub-genotype in males was 3a (80.62%) followed by 1a subtype (3.13%) and 1b subtype (0.98%). Other less frequent subtypes were 2a (0.39%), 3b (0.39%), 2b (0.19%) and 5a (0.19%). In females, most frequent sub-genotypes were 3a (84.7%), 1a (2.06%), 1b (0.61%), 2a (0.20%) and 2b (0.20%). On the other hand sub-genotypes like 3b and 5a were absent in females (Figure 1).

**Table 1:** HCV prevalence in different regions of Punjab province

**Genotype**	**Sub-type**	Districts*							
		**Lahore**	**Faisalabad**	**Gujranwala**	**Gujrat**	**Sialkot**	**Sargodha**	**Mandi Baha-ud-Din**	**Jhang**
1	1a	9* (2.83%)^#^	1(1.42%)	4(3.10%)	2(1.88%)	1(1.06%)	2(3.33%)	5(3.70%)	2(2.32%)
	1b	2(0.63%)	1(1.42%)	1(0.77%)	0	1(1.06%)	1(1.66%)	1(0.74%)	1(1.16%)
2	2a	0	1(1.42%)	0	0	0	1(1.66%)	0	1(1.16%)
	2b	0	1(1.42%)	1(0.77%)	0	0	0	0	0
3	3a	240(75.70%)	60(85.71%)	107(82.94%)	98(92.45%)	76(80.85%)	46(76.66%)	119(88.14%)	76(88.37%)
	3b	0	0	1(0.77%)	0	0	0	0	1(1.16%)
5	5a	0	0	0	0	1(1.06%)	0	0	0
Mixed		4 (1.26%)	0	6(4.65%)	3(2.83%)	2(2.12%)	3(5%)	4(2.96%)	2(2.32%)
Not detected		62(19.55%)	6(8.57%)	9(6.97%)	3(2.83%)	11(11.70%)	7(11.66%)	6(4.44%)	3(3.48%)
Total		317(31.85%)	70(7.03%)	129(12.96%)	106(10.65%)	92(9.24%)	60(6.03%)	135(13.56%)	86(8.64%)

*Number of samples belonging to respective sub-type.

#Percentage contributed by samples in the district.

**Table 2:** HCV viral load in patients from different genders of various regions of Punjab

**Genotype/subtype**	**Viral load**			P value**
	**< 600,000**	**600,000-800,000**	**> 800,000**	
**Genotype 3**	294*	278	252	0.067
**Other genotypes**	71	42	58	
**Male**	199	159	153	0.314
**Female**	166	161	157	
**Lahore**				
**Genotype 3**	79	87	74	0.112
**Other genotypes**	24	20	33	
**Faisalabad**				
**Genotype 3**	24	19	17	0.260
**Other**	4	1	5	
**Gujranwala**				
**Genotype 3**	40	31	37	0.932
**Other**	7	6	8	
**Gujrat**				
**Genotype 3**	31	41	26	0.527
**Other**	4	2	2	
**Sargodha**				
**Genotype 3**	13	18	15	0.397
**Other genotypes**	4	3	7	
**Silakot**				
**Genotype 3**	31	23	22	0.915
**Other**	7	4	5	
**Jhang**				
**Genotype 3**	30	25	22	0.821
**Other**	4	2	3	
**Mandi Baha ud Din**				
**Genotype 3**	53	36	30	0.278
**Other**	10	2	4	

*Number of samples belonging to respective sub-type and under respective viral load.

**Chi Square statistics used to analyse both genotypes and viral loads.

**Figure 2:**
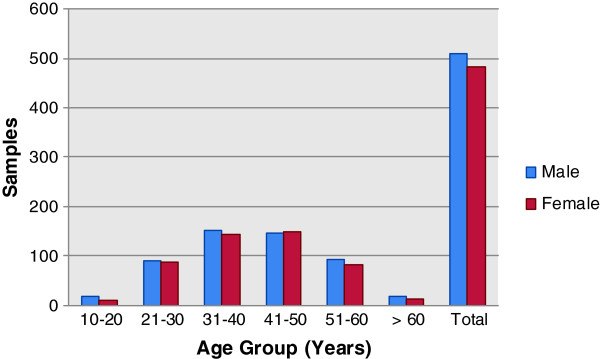
Prevalence of HCV in different age groups.

On basis of age, different groups of genotyped samples were formed such as 10–20 years, 21–30 years, 31–40 years, 41–50 years, 51–60 years and above 60 years. The overall percentage of positive HCV samples in all age groups was higher in males as compare to females except in the age group 41 – 50 years (Figure 2). The distribution of genotypes in all age group categories was also observed. Genotype 3a was highest in all age groups (82.61%) followed by genotypes 1a (2.61%), 1b (0.80%), 2a (0.30%), 2b (0.20%), 3b (0.20%), 5a (0.10%) and mixed genotypes (2.41%).

Among the total HCV positive samples, 317(31.85%) were belonged to Lahore. Among these, 9(2.83%) were genotype 1a, 2(0.63%) were genotype 1b, 240(75.70%) were genotype 3a whereas 62(19.55%) were un-typeable and genotype 4 (1.26%) was with mixed genotypes. The HCV positive patients of Faisalabad were 70(7.03%). Among these, 1(1.42%) belonged to 1a, 1(1.42%) was 1b, 1(1.42%) was 2a, 1(1.42%) was 2b, 60(85.71%) were 3a and 6(8.57%) were un-typeable genotypes. Among the total of 129(12.96%) samples from Gujranwala, 4(3.10%) were 1a, 1(0.77%) was 1b, 1(0.77%) belonged to 2b, 107(82.94%) were 3a, 1(0.77%) was 3b, 9(6.97%) were untypeable genotypes and 6(4.65%) were with mixed genotypes (Table 1).

Prevalence of HCV genotypes among 106(10.65%) positive patients of Gujrat was 2(1.88%) patients were with 1a, 98(92.45%) were with 3a, 3(2.83%) were with mixed infection and 3(2.83%) were with unclassified HCV band. Total 94(9.44%) patients were genotyped from Sialkot in which 1(1.06%) was 1a, 1(1.06%) were 1b, 1(1.06%) was 2a, 76(80.85%) were 3a, 1(1.06%) was 3b, 1(1.06%) was 5a genotype, 2(2.12%) were mixed genotypes and 11(11.70%) were unclassified genotypes. Genotyped samples HCV samples belonged to Sargodha were 60(6.03%). Among these, 2(3.33%) were 1a, 1(1.66%) was 1b, 1(1.66%) was 2a, 46(76.66%) were 3a, 3(5%) were mixed and 7(11.66%) were untypeable genotypes. Total HCV samples belonged to Mandi Baha ud Din were 135(13.56%). Among these, 5(3.70%) were 1a, 1(0.74%) was 1b, 119(88.14%) were 3a and 4(2.96%) were mixed gentoypes. Six 6(4.44%) were untypeable genotypes. The Genotyped samples that belonged to Jhang were 86(8.64%). Among these, 2(2.32%) were 1a, 1(1.16%) were 1b, 1(1.16%) were 2a, 76(88.37%) were 3a, 1(1.16%) was 3b, 2(2.32%) were mixed genotypes and 3(3.48%) were untypeable genotypes (Table 1).

## Discussion

In this study, we demonstrated the HCV genotype distribution in different categories and the existence of association between genotypes and gender. The current study evidently depicts that there is no significant difference between gender and HCV genotypes because HCV genotypes were distributed with equal probability between the both sexes. These results are in accordance with many preceding studies showing no statistical difference in gender with respect to variation among genotypes in Pakistan [11-13]. Current study contradicts with a Libyan study in which HCV genotype 1 possessed significant association with males whereas genotype 4 showed high frequency in females [14]. The results of this study have shown that high prevalence of HCV infection was found at age of <50 years. These findings are in partial agreement with the former studies [10,11,15] that noted the high prevalence in age group of ≤40 years [13,16].

It has been observed that HCV genotype 3a is highly prevalent in Pakistan [10-13,15-17], in contrast, a study has revealed the results from Balochistan where the 1a has higher frequency [7]. Current study revealed that genotype 3a is most frequent and common followed by genotype 1a in HCV infected patients. The HCV genotype distribution pattern is similar to the reported studies from neighbouring South Asian countries where genotype 3 is more frequent than others [16]. Current study has also detected a rare genotype 5a, which is less reported and less frequent. This finding is in agreement with the previous studies that showed the presence of 5a but rare status of this genotype [7,12,15]. These findings are in contradiction with some studies that have shown the partial absent status of genotypes 4, 5, 6 [10].

In this study, we have determined the viral load with respect to genotypes in different districts and gender as showed in Table 2. On the basis of specified levels, we categorized viral load into three classes i.e. low level (< 60, 0000 IU/ml), intermediate level (60,0000-80,0000 IU/ml) and high level (> 80,0000 IU/ml). The results of our study are in line with previous findings that there are no significant differences in HCV RNA levels of both genders (Table 2) [18]. We have observed that the viral load in 29% patients was less than 600,000 IU/mL, while it was between 600,000-800,000 IU/mL in 27.83% patients and 25.22% patients had more than 800,000 IU/mL viral load. It is already established that under therapeutic considerations sustained virological response (SVR) can be easily attained in genotype 3 HCV patients with low viral load (<600,000-800,000 IU/ml) when compared with patients having high viral load (>600000-800000 IU/mL) [19,20]. HCV patients considered potential candidates for Interferon (IFN) therapy. But most of people in Pakistan deprived of this therapy because of high cost, little awareness among common people and lack of genotyping information in many geographical regions. Limited data regarding treatment of HCV is available in Pakistan and no documented evidence has been found on the percentage of patient treated with IFN. So, based on this study a well-planned treatment protocol can be conducted and record of IFN treatment therapy can be carried out.

## Conclusion

Current findings conclude that multiple genotypes of HCV exist in Pakistan in which 3a and 1a are more common. A better understanding of the impact of high HCV RNA levels during the progression of HCV infection is needed, in order to find patients who would get the most benefits from treatment against HCV.

## Competing interests

The authors declare that they have no competing interests.

## Author’s contributions

SQA, MNZ and TY designed and supervised research. SQA, MMA, FA, IQA, SQA collected the samples and performed experiments. MMA, FA and MNZ analysed the data. SQA, FA, and MMA drafted the manuscript and all authors read and approved the final manuscript.
